# Evaluating the Mainstream Influence of Cardiothoracic Surgical Research: An Analysis of Google Trends Data from 2004 to 2024

**DOI:** 10.21470/1678-9741-2025-0146

**Published:** 2026-06-08

**Authors:** H Shafeeq Ahmed

**Affiliations:** 1 Bangalore Medical College and Research Institute, Bengaluru, Karnataka, India

**Keywords:** Cardiovascular Surgical Procedures, Thorax, Thoracic Surgery, Cardiovascular Disease, Patient Education, Information Science.

## Abstract

**Introduction:**

Public awareness and interest significantly influence research priorities and healthcare advancements. This study investigates the relationship between public interest, represented by Google Trends Relative Search Volume (RSV), and cardiothoracic research outputs over 21 years (2004 - 2024).

**Methods:**

A total of 26 conditions/surgeries representing eight topics of general cardiothoracic interest were identified from a review of various social media pages, society webpages, and hospital information bulletins. Data on the conditions were collected from Google Trends and PubMed®. RSV values were calculated annually, and publication counts were extracted for each condition. The study used R (v4.3.3) for all statistical analyses and predictive models.

**Results:**

Trauma-related conditions and extracorporeal membrane oxygenation (ECMO) demonstrated increasing RSV and publication trends, with strong positive correlations (e.g., ECMO: r = 0.88, P < 0.0001). Conditions such as congenital cardiac anomalies (e.g., tetralogy of Fallot: r = -0.74, P < 0.0001) showed a negative correlation, with declining RSV despite ongoing research. Multiple regression revealed a significant positive relationship between RSV and publication counts when conditions were controlled (slope = 16.68, R^2^ = 0.8081, P < 0.0001). Feature importance analysis showed that "Condition" had a slightly greater influence than RSV on publication trends.

**Conclusion:**

The study demonstrates variability between public interest and research output across cardiothoracic conditions. While some conditions, such as trauma-related cases and ECMO, show alignment between public awareness and publication activity, others, including congenital anomalies, exhibit divergence.

## INTRODUCTION

**Table:** 

Abbreviations, Acronyms & Symbols				
AA	= Aortic aneurysm		LT	= Lung transplant
ANOVA	= Analysis of variance		M	= Mesothelioma
B	= Bronchiectasis		MARS	= Multivariate Adaptive Regression Splines
CABG	= Coronary artery bypass grafting		MED	= Mediastinitis
CI	= Confidence interval		P	= Pacemaker
EA	= Ebstein’s anomaly		PC	= Pectus carinatum
EC	= Esophageal cancer		PE	= Pectus excavatum
ECMO	= Extracorporeal membrane oxygenation		PN	= Pneumothorax
EsA	= Esophageal achalasia		RF	= Rib fracture
FC	= Flail chest		RSV	= Relative Search Volume
GERD	= Gastroesophageal reflux disease		SVCS	= Superior vena cava syndrome
HLHS	= Hypoplastic left heart syndrome		T	= Thymoma
HSD	= Honestly Significant Difference		TGA	= Transposition of great arteries
HT	= Heart transplant		TOF	= Tetralogy of Fallot
LA	= Lung abscess		TOS	= Thoracic-outlet syndrome
LC	= Lung cancer			

Cardiovascular diseases, including cardiothoracic conditions, are the leading cause of morbidity and mortality worldwide, accounting for a significant proportion of deaths annually^[1]^. Despite advances in prevention, diagnosis, and treatment, the prevalence of cardiothoracic conditions, such as coronary artery disease, valvular heart disease, and aortic aneurysms, continues to rise due to aging populations and lifestyle factors^[2,3]^. Cardiothoracic surgical interventions, ranging from minimally invasive techniques to complex open-heart procedures, have played a pivotal role in managing severe cases^[4]^. However, disparities in awareness, access, and the public understanding of these conditions pose challenges to optimal healthcare delivery^[5]^.

Public awareness and interest significantly influence healthcare decision-making, funding allocation, and research priorities^[6]^. Online search trends and public queries serve as a proxy for understanding public concerns and gaps in knowledge about health conditions^[7,8]^. While the volume of cardiothoracic research publications has increased over the years, analyzing correlations between public interest and research output can enhance communication strategies, ensuring that advancements in the field reach wider audiences. Informing the public about new developments in cardiothoracic surgery and correlating research outputs with public attention can facilitate broader dissemination of knowledge and promote understanding.

Engaging the public in discussions about cardiothoracic diseases and aligning research with public health priorities can help garner interest and, subsequently, increase funding for related studies^[9,10]^. Public concerns about health issues often shape policy priorities and influence resource allocation. This study aims to analyze 21 years of Google Trends data alongside PubMed® publication trends to explore the evolving public interest in cardiothoracic conditions and their potential connections to research outputs. By identifying trends and correlations, this study seeks to highlight opportunities for enhancing public awareness and ensuring that research contributions address pressing public health issues, ultimately fostering greater societal and institutional support.

## METHODS

The present study utilizes two primary sources to evaluate progress in cardiothoracic research and public interest: PubMed® and Google Trends. The study was conducted in January 2025. As data was collected from freely available data (https://trends.google.com/trends/), institutional ethical approval was not required.

### Data Collection

Google Trends provides statistical data on search queries. Users can input specific terms of interest, select a region (global or a specific country), and define a time frame (starting from January 2004). The platform generates a time series chart based on a 1% sample of all searches. The data is presented as Relative Search Volume (RSV), scaled from 0 to 100, where 0 indicates no recorded interest (0%) and 100 represents the highest level of interest (100%) within the selected time and location. RSV values are normalized to account for the number of Google users in the specified period and region. In this study, monthly readings of global data between January 2004 and December 2024 were acquired for each search term.

Google Trends is a valid proxy for evaluating public interest in healthcare and allied fields. The present study follows the recommendations of Nuti et al.^[11]^ to ensure the transparency, reproducibility, and quality of the study’s methods.

### Search Terms

**Fig. 1:**
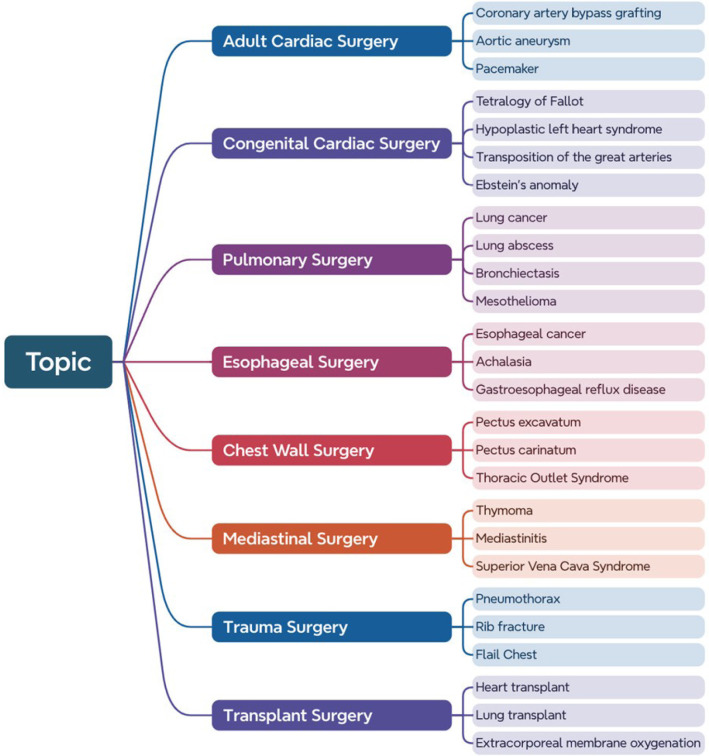
Flowchart of the various conditions included in the study.

To identify common cardiothoracic conditions queried by the public, a comprehensive evaluation of online sources was performed. This included analyzing websites of major professional cardiothoracic, thoracic, and cardiological societies (for instance, Society for Cardiothoracic Surgery in Great Britain & Ireland and Society of Thoracic Surgeons) as well as international hospital websites. The identified conditions were categorized into eight main groups (hereafter simply referred to as “topic(s)”), each including a range of related conditions (full classification of included conditions is available in Figure 1). It is important to note that a few of the included conditions are not always treated by cardiothoracic surgeons but rather through a multidisciplinary approach involving different specialties. However, their inclusion was deemed critical, as it allowed for a more comprehensive understanding of the role of cardiothoracic surgery and other allied specialties, and provided a holistic view. Given the focus on public interest, the study primarily references conditions rather than specific surgical procedures. For each category, three to four of the most commonly mentioned conditions were finalized, resulting in a total of 26 unique conditions or surgeries (hereafter simply referred to as “condition(s)”) being identified.

The data from PubMed® was extracted for each condition using specific search terms combined with Boolean operators to create a tailored search string for each individual condition. The complete list of search terms for each condition is provided in Supplementary Table 1.

**Table 1:** Pearson Correlation Results.

Condition	Coefficient R	*P*-value
CABG	-0.169	0.46
Aortic aneurysm	0.427	0.053
Pacemaker	0.521	0.015
Tetralogy of Fallot	-0.745	0.0001
Hypoplastic left heart syndrome	-0.724	0.0002
Transposition of the great arteries	-0.525	0.014
Ebstein’s anomaly	-0.676	0.0007
Lung cancer	-0.677	0.0007
Lung abscess	0.694	0.0004
Bronchiectasis	0.871	< 0.0001
Mesothelioma	-0.320	0.15
Esophageal cancer	-0.307	0.17
Achalasia	0.761	< 0.0001
Gastroesophageal reflux disease	0.924	< 0.0001
Pectus excavatum	-0.407	0.066
Pectus carinatum	0.635	0.0019
Thoracic outlet syndrome	0.254	0.26
Thymoma	-0.436	0.048
Mediastinitis	-0.429	0.052
Superior vena cava syndrome	-0.205	0.37
Pneumothorax	0.858	< 0.0001
Rib fracture	0.797	< 0.0001
Flail chest	0.858	< 0.0001
Heart transplant	0.055	0.81
Lung transplant	0.035	0.87
Extracorporeal membrane oxygenation	0.883	< 0.0001

CABG=coronary artery bypass grafting

### Data Analysis

Data was extracted and filled into a pre-piloted spreadsheet. PubMed® data was obtained year-wise, and Google Trends data was averaged annually by calculating the mean RSV across the 12 months of each year. All data analysis was conducted using R version 4.4.2. RSV data was plotted against time for each topic to understand condition-wise variations for each topic. A one-way analysis of variance (ANOVA) and subsequent post-hoc Tukey Honestly Significant Difference (HSD) analyses were done to compare the variations in RSV across and between conditions. Similarly, annual publication counts were plotted against time. Subsequently, scatter plots for each of the eight topics were generated, including convex hulls for each topic and linear regression lines with confidence intervals (CIs). Additionally, an overall plot was created to assess trends and correlations comprehensively. A Pearson correlation was performed to evaluate the influence of RSV on the publication count. Subsequently, multiple linear regression analysis was performed to evaluate the combined influence of RSV and conditions on publication counts. Model evaluation was performed, including analysis of residuals and outliers to assess model fit. The Multivariate Adaptive Regression Splines (MARS) approach was used to model the complex, non-linear relationships between RSV and the number of publications. Following the MARS analysis, a Random Forest model was used to further investigate the proportionate influence of conditions (as categorical variables) and RSV (as a continuous variable) on the number of publications.

## RESULTS

**Fig. 2:**
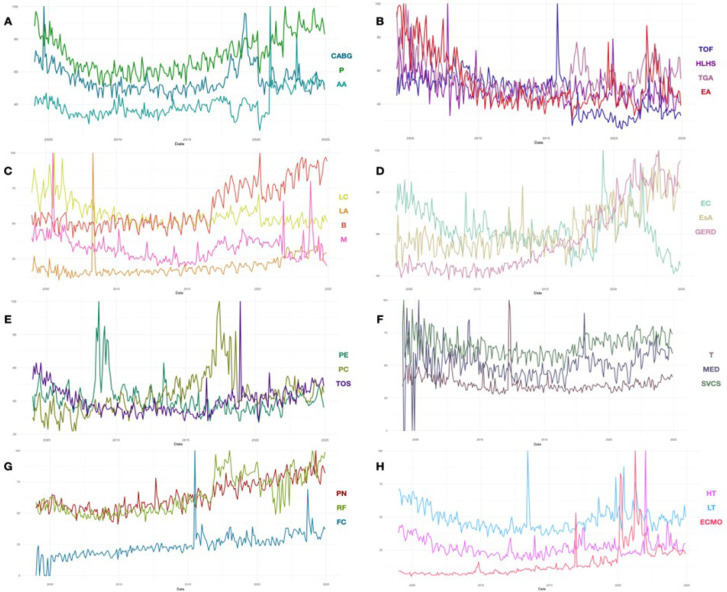
Changes in Relative Search Volume monthly from 2004 to 2024 for (A) adult cardiac surgeries; (B) congenital cardiac surgeries; (C) pulmonary surgeries; (D) esophageal surgeries; (E) chest wall surgeries; (F) mediastinal surgeries; (G) trauma surgeries; and (H) transplant surgeries. AA=aortic aneurysm; B=bronchiectasis; CABG=coronary artery bypass grafting; EA=Ebstein’s anomaly; EsA=esophageal achalasia; EC=esophageal cancer; ECMO=extracorporeal membrane oxygenation; FC=flail chest; GERD=gastroesophageal reflux disease; HLHS=hypoplastic left heart syndrome; HT=heart transplant; LA=lung abscess; LC=lung cancer; LT=lung transplant; M=mesothelioma; MED=mediastinitis; P=pacemaker; PC=pectus carinatum; PE=pectus excavatum; PN=pneumothorax; RF=rib fracture; SVCS=superior vena cava syndrome; T=thymoma; TGA=transposition of great arteries; TOF=tetralogy of Fallot; TOS=thoracic-outlet syndrome.

**Fig. 3:**
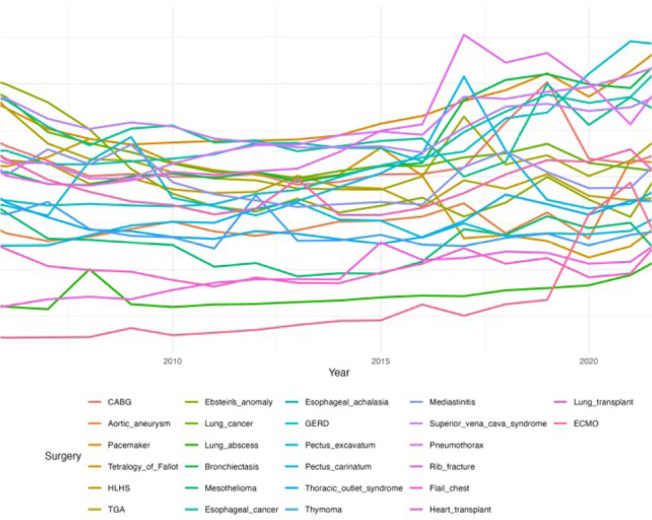
Average annual condition-wise Relative Search Volume trends. CABG=coronary artery bypass grafting; ECMO=extracorporeal membrane oxygenation; GERD=gastroesophageal reflux disease; HLHS=hypoplastic left heart syndrome; TGA=transposition of great arteries.

The study analyzed 26 conditions categorized into eight topics. Figure 2 illustrates the topic-wise changes in RSV data over the years. From 2004 to 2024, the trends indicate an increase in interest for conditions such as pacemaker implantation, bronchiectasis, esophageal achalasia, gastroesophageal reflux disease (GERD), all trauma-related conditions, and extracorporeal membrane oxygenation (ECMO). Conversely, a decline in interest was observed for all congenital cardiac conditions, lung cancer, mesothelioma, and esophageal cancer. Other conditions showed relatively minor variations in interest over the period analyzed. A one-way ANOVA revealed a significant effect of conditions on RSV (F [25, 520] = 51.7, *P* < 0.0001). Tukey HSD post-hoc test revealed differences in trends between various condition pairs (detailed Tukey HSD results can be found in Supplementary File 1). Figure 3 shows the overall average annual trends in RSV across the topics. On the other hand, Supplementary Figure 1A shows the annual changes in publication counts, with Supplementary Figure 1B showing this specifically for lung cancer, as the large count of this condition affected the readability of the overall chart.

**Fig. 4:**
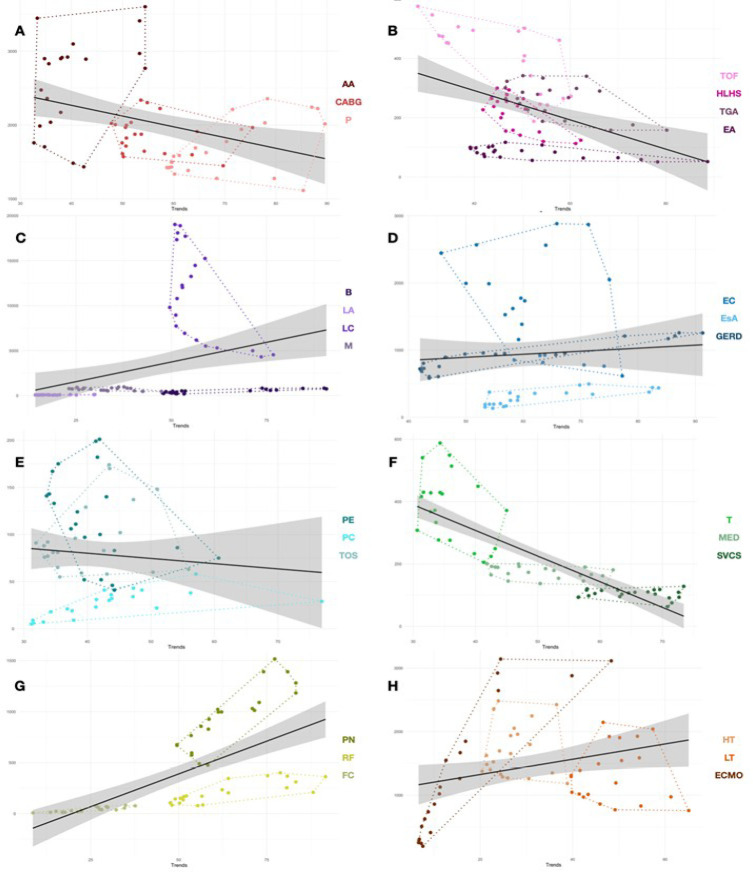
Scatterplot with convex hulls and regression lines for Relative Search Volume against publication counts annually from 2004 to 2024 for (A) adult cardiac surgeries; (B) congenital cardiac surgeries; (C) pulmonary surgeries; (D) esophageal surgeries; (E) chest wall surgeries; (F) mediastinal surgeries; (G) trauma surgeries; and (H) transplant surgeries. AA=aortic aneurysm; B=bronchiectasis; CABG=coronary artery bypass grafting; EA=Ebstein’s anomaly; EsA=esophageal achalasia; EC=esophageal cancer; ECMO=extracorporeal membrane oxygenation; FC=flail chest; GERD=gastroesophageal reflux disease; HLHS=hypoplastic left heart syndrome; HT=heart transplant; LA=lung abscess; LC=lung cancer; LT=lung transplant; M=mesothelioma; MED=mediastinitis; P=pacemaker; PC=pectus carinatum; PE=pectus excavatum; PN=pneumothorax; RF=rib fracture; SVCS=superior vena cava syndrome; T=thymoma; TGA=transposition of great arteries; TOF=tetralogy of Fallot; TOS=thoracic-outlet syndrome.

Figure 4 shows a scatterplot with convex hulls to identify the correlation between number of publications and RSV. A negative relationship between RSV and the number of publications was observed for congenital and adult cardiac surgeries, as well as mediastinal surgeries. A very mild decrease was noted for chest wall surgeries, although the CI for the regression line was very wide. On the other hand, all other procedures showed positive trends in both the number of publications and increasing RSV. In particular, a notable positive effect was observed for trauma surgeries.

Table 1 shows the results of the Pearson correlation analysis between RSV and number of publications for each of the individual conditions. Tetralogy of Fallot (r = -0.74, *P* < 0.0001) and hypoplastic left heart syndrome (r = -0.72, *P* = 0.0002) showed significant negative correlations with RSV. On the other hand, bronchiectasis (r = 0.87, *P* < 0.0001) and ECMO (r = 0.88, *P* < 0.0001) showed a highly significant positive correlation. Many other conditions showed significant positive and negative correlations.

**Supplementary Figure 2:**
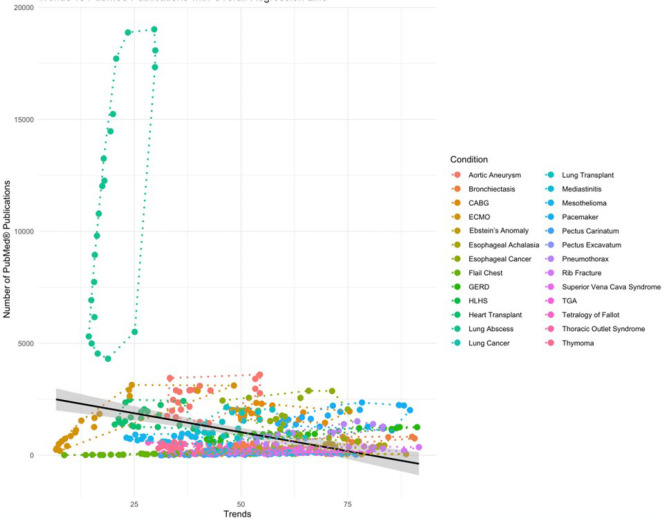
Relationship between relative search volume and publication counts across cardiothoracic conditions. CABG=coronary artery bypass grafting; ECMO=extracorporeal membrane oxygenation; GERD=gastroesophageal reflux disease; HLHS=hypoplastic left heart syndrome; TGA=transposition of great arteries.

The initial simple linear regression analysis (Supplementary Figure 2) revealed a negative relationship between RSV and publication counts (slope = -33.82, *P* < 0.0001), explaining only 6.31% of the variance (R^2^ = 0.0631). To better account for variability, a multiple regression analysis was conducted, incorporating both RSV and conditions as predictors. This model demonstrated a positive relationship between RSV and publication counts (slope = 16.68, *P* < 0.0001) and explained 80.8% of the variance (R^2^ = 0.8081), with minimal multicollinearity (variance inflation factor for RSV = 1.867; Conditions = 1.025). Additionally, MARS modeling revealed a modest but threshold-dependent relationship between RSV and publication counts (R^2^ = 0.1463), with publications decreasing at RSV values below 23.92 (β = -149.51) and increasing above 43 (β = 167.75). To further explore variability in publication counts, Random Forest analysis indicated that “Conditions” had a slightly greater influence than RSV (the model accounted for 79.02% of the variance).

## DISCUSSION

The present study employs advanced statistical methods for a robust evaluation of variations in Google Trends data (main component: RSV) compared with the number of publications (year-wise data acquired from PubMed®) to identify potential correlations across 26 cardiothoracic conditions. This study provides insights into public interest trends over the past 20 years (2004 - 2024) for various conditions and examines whether these trends influence research outputs. The study noted a strong positive correlation between research output and public interest, with the overall number of publications increasing by approximately 16 papers for a unit rise in RSV. However, significant variability was noted across conditions, with Pearson correlation analyses showing notable differences. Some conditions exhibited apparent correlations, indicating a potential misalignment between public interest and research focus^[12]^. This discontinuity indicates that, for certain conditions, rising public interest is associated with fewer publications, and vice versa. Conversely, for other conditions, there is a proportional relationship where increased public interest corresponds with a rise in research output, and decreased interest correlates with a decline in research activity.

The results indicate a growing positive influence on both research output and public interest for trauma-related conditions, as well as for other conditions like ECMO, which are driven by technological advancements. Conversely, conditions such as achalasia and GERD are influenced by the increasing prevalence of health dysfunctions and lifestyle disorders, reflecting the impact of modern lifestyles over the past few years on the growing incidence of these conditions^[13,14]^. In contrast, congenital cardiac conditions and cancer-related conditions show a negative correlation. This trend may be attributed to a decline in public interest despite an increase in research output^[15]^. Changes in healthcare policy, educational efforts, and the influence of media coverage are all aspects that impact interest in congenital cardiac conditions. Improvements in obstetric care and the use of fetal echocardiography are also advancements that allow clinicians to catch anomalies at an earlier stage^[16]^. This indicates a critical need to educate and inform the public about these conditions, as they are not only critical but also require early detection for effective management, particularly in the case of congenital cardiac anomalies and cancers.

This misalignment between public interest and research output is a critical issue that requires urgent attention in the context of cardiothoracic surgery. In a field that has been under-researched and understudied in recent years compared to other specialties, aligning research output with public interest needs to be strategically managed^[17,18]^. While rising public attention, as reflected by RSV, can highlight areas of societal concern, research strategy should not be dictated solely by popularity. Scientific priorities must continue to emphasize clinical urgency, unmet medical needs, and evidence-based importance^[19]^. Nevertheless, public engagement remains valuable: targeted education campaigns can raise awareness about critical but under-recognized conditions, such as congenital cardiac anomalies and cancers, potentially fostering informed interest and supporting long-term research sustainability^[20]^. Both approaches, prioritizing urgent clinical needs while enhancing public awareness, are essential for optimizing research impact and societal benefit in cardiothoracic surgery.

The interests of the public do not appear to be consistently aligned with publication counts, as there is considerable variability observed in this study. Notably, a threshold-based pattern emerges: publication counts tend to rise when RSV exceeds 43, while a negative impact is observed when RSV falls below 23. This suggests that the correlation between RSV and publication counts is significant and could influence our understanding of how RSV may affect publication trends and vice versa^[21]^. This bilateral relationship between RSV and publication counts is particularly intriguing and warrants further investigation for a more comprehensive understanding. However, the findings of this study alone are insufficient to draw definitive conclusions.

It is important to note that the use of Google Trends to track public interest is not a novel concept. While this study focuses specifically on cardiothoracic surgery, other researchers have applied similar methodologies in different contexts. For example, Kamiński et al.^[22]^ used Google Trends data to identify public interest trends related to various surgical and pharmacological treatments for obesity. Their study highlighted how public interest may sometimes be misguided or focused on unconventional or unapproved drugs, demonstrating the potential of Google Trends to track public misconceptions. Similarly, Han et al.^[23]^ explored this approach in the context of glucagon-like peptide-1, further illustrating the utility of Google Trends in healthcare research. During the coronavirus disease 2019 pandemic^[24]^, this method has also proven valuable for monitoring and predicting disease outbreaks, as well as in other conditions for forecasting regions with potential outbreak surges such as monkeypox^[25]^. These studies highlight the effectiveness of using Google Trends to evaluate and enhance public understanding of diseases through targeted educational measures. However, while this study and others use RSV as a quantifiable measure of public interest, this metric reflects attention rather than scientific or clinical value, and correlations with research output may risk emphasizing popularity over clinically critical but less visible conditions.

### Strengths and Limitations

The present study has several strengths, including its exploration of diverse cardiothoracic conditions while accounting for variability and depth. It also utilizes advanced statistical modeling techniques to ensure the accuracy and robustness of the findings. The study identifies several key points that may be critical in informing a large proportion of the cardiothoracic sector about the path ahead. However, despite its strengths, there are limitations inherent to such studies. Firstly, the conditions analyzed are not exclusively to those treated by cardiothoracic surgeons but also involve a wide range of other healthcare professionals, including but not limited to cardiologists, pulmonologists, oncologists, gastroenterologists, general surgeons, internists, pediatric surgeons, and others. Additionally, the strategies employed are simple and limited to the English language, which may have resulted in fewer related conditions being captured. Addressing this complexity would be time-consuming and beyond the scope of this paper. Furthermore, while the correlation of the RSV with the number of publications demonstrates a linear relationship, it also reveals a threshold-based understanding that may complicate interpretation. However, this limitation is mitigated to some extent by the accountability of the multilinear regression models and apparent models, which show high concurrence with linearity. The final and most significant limitation is the presence of potential confounders that may influence public opinion (including but not limited to media bias, fake news, celebrities, etc.). These confounders are challenging to address within the scope of this study. Nonetheless, their influence may not necessarily be significant, considering the non-linear fluctuations in RSV.

### Implications and Future Directions

The findings of this study highlight the complex relationship between public interest, as measured by RSV, and research output in cardiothoracic conditions. One actionable implication is the potential for targeted public education campaigns. For conditions such as congenital cardiac anomalies and cancers, which show a disconnect between public interest and research efforts, initiatives to raise awareness could foster greater public engagement and stimulate interest in related fields. This goes beyond simply encouraging research but is also critical in the context of public health education and empowering patients. Public health initiatives, in collaboration with family medicine specialists, can play a pivotal role in encouraging community interest in healthcare, particularly in addressing critical health conditions such as cancers. They should also emphasize not only responding to public interest but also fostering scientific responsibility by raising awareness of underrepresented yet clinically critical conditions. Furthermore, the strong positive correlations for trauma-related conditions and ECMO suggest that technological advancements and emergent public health needs are influential factors driving both interest and research.

Future strategies should focus on understanding these trends and factors that affect them, investing in fields showing rapid growth, and addressing underrepresented conditions that are critical to improving patient outcomes. In practical terms, policymakers, funding agencies, and research institutions can use these insights to better allocate resources. For example, prioritizing research funding in areas with demonstrated public interest, such as trauma surgery and lifestyle issues like achalasia and GERD, can sustain the momentum in these fields. Conversely, conditions with declining public interest but significant clinical importance, such as congenital cardiac conditions, should be supported through long-term funding and strategic collaborations to ensure sustained research efforts. Finally, inculcating the use of integrative modeling tools in research prioritization can help predict trends and optimize the impact of future studies on cardiothoracic health outcomes.

## CONCLUSION

This study provides critical insights into the evolving dynamics between public interest and research output in cardiothoracic surgery over the past two decades. By identifying patterns of growth, stagnation, and decline in specific conditions, we can identify areas of both alignment and disparity between societal concerns and academic focus. The findings reveal a strong influence of public interest on research productivity in certain areas, while also pointing to gaps where interest and output diverge. For the field of cardiothoracic surgery as a whole, this study underscores the importance of strategically aligning research initiatives with public health needs and trends. Doing so will not only enhance the relevance of research but also contribute to better-informed public awareness and improved patient outcomes in this critical specialty.

## Figures and Tables

**Supplementary Figure 1A:**
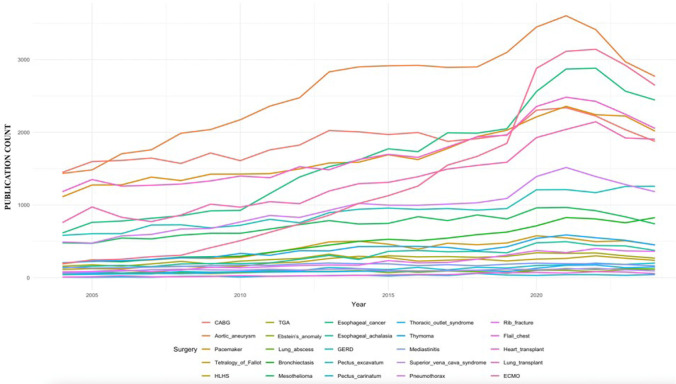
Temporal trends in cardiothoracic surgical publications from 2004 to 2024. CABG=coronary artery bypass grafting; ECMO=extracorporeal membrane oxygenation; GERD=gastroesophageal reflux disease; HLHS=hypoplastic left heart syndrome; TGA=transposition of great arteries.

**Supplementary Figure 1B:**
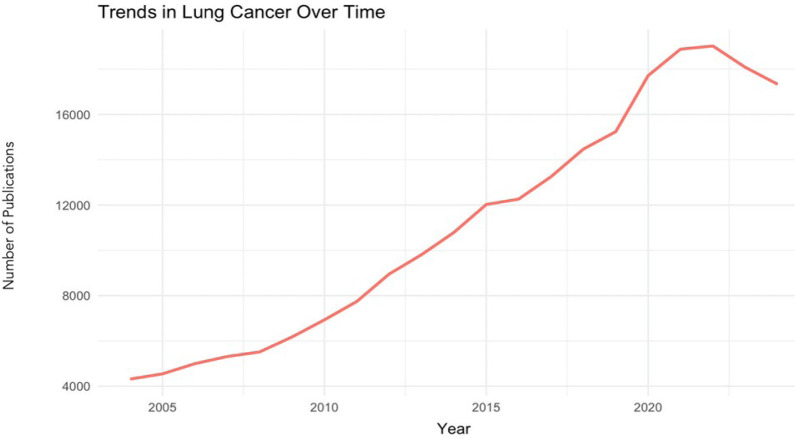
Temporal trends in lung cancer publications from 2004 to 2024.

## Data Availability

The author declares that the Supplementary Table 1 and Supplementary File 1 are openly available in the Mendeley Data repository at https://data.mendeley.com/datasets/kx6mr8bd5v/1 (DOI: 10.17632/kx6mr8bd5v.1).

## Artificial Intelligence Usage

The author declares use of Grammarly for corrections. The content produced by the artificial intelligence tool was revised and edited by the author as necessary, and he takes full responsibility for the content to be published.

## Supplementary Materials

Supplementary Table 1 Condition-wise PubMed® search strategy (this Supplementary Table is openly available in the Mendeley Data repository at https://data.mendeley.com/datasets/kx6mr8bd5v/1 [DOI: 10.17632/kx6mr8bd5v.1]).
